# Cost-effectiveness of first line nivolumab-ipilimumab combination therapy for advanced non-small cell lung cancer: A systematic review and methodological quality assessment

**DOI:** 10.3389/frhs.2023.1034256

**Published:** 2023-03-13

**Authors:** Remziye Zaim, W. Ken Redekop, Carin A. Uyl-de Groot

**Affiliations:** Erasmus School of Health Policy & Management, Erasmus University Rotterdam, Rotterdam, Netherlands

**Keywords:** cost-effectiveness, nivolumab, ipilimumab, non-small cell lung cancer, first-line treatment, systematic review, quality assessment

## Abstract

To assess the methodological quality of cost-effectiveness analyses (CEA) of nivolumab in combination with ipilimumab, we conducted a systematic literature review in the first-line treatment of patients with recurrent or metastatic non-small cell lung cancer (NSCLC), whose tumors express programmed death ligand-1, with no epidermal growth factor receptor or anaplastic lymphoma kinase genomic tumor aberrations. PubMed, Embase, and the Cost-Effectiveness Analysis Registry were searched, in accordance with the Preferred Reporting Items for Systematic Reviews and Meta-Analyses guidelines. The methodological quality of the included studies was assessed by the Philips checklist and the Consensus Health Economic Criteria (CHEC) checklist. 171 records were identified. Seven studies met the inclusion criteria. Cost-effectiveness analyses differed substantially due to the applied modeling methods, sources of costs, health state utilities, and key assumptions. Quality assessment of the included studies highlighted shortcomings in data identification, uncertainty assessment, and methods transparency. Our systematic review and methodology assessment revealed that the methods of estimation of long-term outcomes, quantification of health state utility values, estimation of drug costs, the accuracy of data sources, and their credibility have important implications on the cost-effectiveness outcomes. None of the included studies fulfilled all of the criteria reported in the Philips and the CHEC checklists. To compound the economic consequences presented in these limited number of CEAs, ipilimumab's drug action as a combination therapy poses significant uncertainty. We encourage further research to address the economic consequences of these combination agents in future CEAs and the clinical uncertainties of ipilimumab for NSCLC in future trials.

## 1. Introduction

Platinum-based doublet chemotherapy was historically the standard first-line treatment for patients with recurrent or metastatic non-small cell lung cancer (NSCLC), whose tumors lack epidermal growth factor receptor mutations or anaplastic lymphoma kinase translocations. More recently, pembrolizumab monotherapy for patients with a high level of tumor programmed cell death ligand-1 (PD-L1) expression ≥1% became the standard first-line therapy for advanced NSCLC without treatable driver mutations (1–3). Nivolumab and ipilimumab are monoclonal antibodies that bind to programmed death-1 (PD-1) and cytotoxic T-lymphocyte antigen 4 (CTLA-4) receptors, respectively, to restore T-cell activity against tumor cells. In 2019, the CheckMate 227 Phase 3 trial showed improved progression-free and overall survival with this dual checkpoint inhibition in recurrent or metastatic NSCLC (4). The CheckMate 227 trial results indicated that nivolumab in combination with ipilimumab was associated with improved survival in pre-specified subgroups, including PD-L1 ≥ 1% and PD-L1 < 1% (4). In 2021, the CheckMate 9LA Phase 3 trial, stratified patients by PD-L1 ≥ 1% and <1%, showed that nivolumab in combination with ipilimumab plus two cycles of chemotherapy improved progression-free and overall survival, compared with four cycles of chemotherapy (5). The United States (US) Food and Drug Administration (FDA) approved nivolumab in combination with ipilimumab for patients with PD-L1 ≥ 1% (6), and the National Comprehensive Cancer Network panel extended their use for patients with PD-L1 < 1% (7). Nivolumab plus ipilimumab with two cycles of chemotherapy was also approved by the US FDA for patients regardless of PD-L1 expression levels (8).

Although several studies have shown single-agent immune checkpoint inhibitors with or without chemotherapy to be cost-effective (9–13), double-agent immunotherapy combinations may not be deemed cost-effective, given their high price tags. To assess the economic value of nivolumab in combination with ipilimumab, we conducted a systematic literature review of model-based cost effectiveness analyses (CEA) in the first-line treatment of patients with recurrent or metastatic NSCLC. To evaluate the methodological quality of the published CEAs, we used the Philips checklist (14), and the Consensus Health Economic Criteria (CHEC) checklist (15), to critically review the applied methods and modelling efforts in this setting.

## 2. Methods

### 2.1. Search strategy

A systematic literature review was conducted in accordance with the Preferred Reporting Items for Systematic Reviews and Meta-Analyses (PRISMA) guidelines (16). We searched PubMed, Embase, and the Cost-Effectiveness Analysis (CEA) Registry database. The searches were built using the Population Intervention Comparison Outcome (PICO) framework (Supplementary Tables S1A–C). Each search was limited to English-language studies of human subjects. No date restrictions were applied. The search strategy included *MeSH* terms in PubMed and *Emtree* terms in Embase, as well as free-text terms in the CEA Registry database (Supplementary Table S1). Manual reference checks supplemented database searches. All searches were finalized on January 5, 2022.

### 2.2. Study selection

Studies accepted at the title-abstract screening stage were retrieved in full text for review. Two reviewers screened all studies and resolved any issues of discrepancy through consensus or consultation with a third reviewer. Studies were included if they fulfilled the eligibility criteria. The process of selection and inclusion and exclusion of articles was recorded in both Rayyan (https://www.rayyan.ai/cite) and Microsoft Excel. This method provides transparency regarding all selection steps and assures reproducibility. The details of the inclusion and exclusion criteria are presented in Table 1.

**Table 1:** Study inclusion and exclusion criteria.

Item	Inclusion	Exclusion
Period publication	No restriction	–
Country of study	Worldwide	–
Study design/type	Cost effectiveness analysis Cost utility analysis	Resource use, patient reported outcomes
Study population	First-line (treatment naïve) metastatic or advanced NSCLC without treatable driver mutations	Any other population
Study intervention	Nivolumab Ipilimumab	All other study interventions
Study comparison	Chemotherapy	No comparator
** **Study outcomes	Quality adjusted life years Incremental Cost Effectiveness Ratio	**–**

NSCLC, Non-small cell lung cancer.

### 2.3. Data extraction

An evidence table (Table 2) is created according to the PICO framework to extract data on the study author, year, country, population, clinical trial, PD-L1 expression, intervention, comparator, time horizon, study perspective, incremental outcomes (QALYs and costs), incremental cost-effectiveness ratio (ICER), as well as the author's stated conclusions.

**Table 2:** Evidence table of the included cost-effectiveness studies.

Cost effectiveness studies of nivolumab-ipilimumab for the first-line treatment of advanced NSCLC										
Author, Year, Country	Population, Clinical Trial	PD-L1 Test	Intervention	Comparator	Time Horizon	Study Perspective	Incremental Outcomes	Incremental Cost-Effectiveness Ratio (ICER)		Conclusions of the study authors
							ΔCosts (US dollars)	ΔQALY	ΔCosts/ΔQALY gained	
**Courtney et al., 2021, (17) United States**	First-line advanced NSCLC, Checkmate 227	PD-L1 ≥ 50%, PD-L1 ≥ 1%, PD-L1 < 1%	Nivo (3 mg/kg Q2W) + Ipi (1 mg/kg Q6W)	Chemo	10 years	US Healthcare and Societal Perspectives	$201,900	0.50	Healthcare: $401,700 per QALY gained Societal: $434,400 per QALY gained	Nivo + Ipi combination therapy was not cost-effective compared with chemo as first-line treatment for patients with advanced NSCLC
**Hao et al., 2021, (18) United States & China**	First-line advanced NSCLC, Checkmate 227	PD-L1 < 1%	Nivo (3 mg/kg Q2W) + Ipi (1 mg/kg Q6W)	Chemo	10 years	US Payer (Medicare & Medicaid) Perspective and Chinese Healthcare Perspective	US: $95,617, China: $66,178	US: 1.26, China: 1.1	US: $75,871 per QALY gained, China: $59,773 per QALY gained	Nivo + Ipi was a cost effective option compared with chemo in the US. However, it was not cost-effective in China.
**Hu et al., 2020, (19) United States**	First-line advanced NSCLC, Checkmate 227	PD-L1 ≥ 50%, PD-L1 ≥ 1%, PD-L1 < 1%	Nivo (3 mg/kg Q2W) + Ipi (1 mg/kg Q6W)	Chemo	20 years	US Payer (Medicare & Medicaid) Perspective	PD-L1 ≥ 50%: $124,181, PD-L1 ≥ 1%: $70,951, PD-L1 < 1%: $144,093	PD-L1 ≥ 50%: 1.15, PD-L1 ≥ 1%: 0.53, PD-L1 < 1%: 0.84	PD-L1 ≥ 50%: $107,404 per QALY gained, PD-L1 ≥ 1%: $133,732 per QALY gained, PD-L1 < 1%: $172,589 per QALY gained	Nivo + Ipi was a better cost-effective strategy than chemo in patients with PD-L1 ≥ 50% and ≥1% or a high TMB, but not in patients with a PD-L1 < 1%, at a WTP of $150 K per QALY gained.
**Li et al., 2020, (20) United States**	First-line advanced NSCLC, Checkmate 227	PD-L1 ≥ 1%, PD-L1 < 1%	Nivo (3 mg/kg Q2W) + Ipi (1 mg/kg Q6W)	Chemo	10 years	US Payer (Medicare & Medicaid) Perspective	PD-L1 < 1% $128,250, PD-L1 ≥ 1% $128,984	PD-L1 < 1% 0.894, PD-L1 ≥ 1% 0.715	PD-L1 < 1% $143,434, PD-L1 ≥ 1% $180,307 per QALY gained	Nivo + Ipi is cost-effective in the first-line setting only in patients with PD-L1 < 1%, but not cost effective for PD-L1 ≥ 1% or for all population.
**Peng et al., 2021, (21) United States**	First-line advanced NSCLC, Checkmate 9LA	Not reported	Nivo (360 mg Q3W) + Ipi (1 mg/kg Q6W) + 2 cycles of chemotherapy	Chemo	Lifetime Horizon	US Payer (Medicare & Medicaid) Perspective	$161,993	0.8	$202,275 per QALY gained	Nivo + Ipi combined with two cycles of chemo was unlikely to be cost-effective as a first-line treatment at the WTP of 150 K per QALY gained.
**Wan et al., 2021, (22) United States**	First-line advanced NSCLC, CheckMate 227	PD-L1 ≥ 50%, PD-L1 ≥ 1%, PD-L1 < 1%	Nivo (3 mg/kg Q2W) + Ipi (1 mg/kg Q6W)	Chemo	Lifetime Horizon	US Payer (Medicare & Medicaid) Perspective	$66,218	0.62	$104,238 per QALY gained	Nivo + Ipi was cost-effective compared with chemo at a WTP threshold between 100 K and 150 K per QALY gained.
**Yang et al., 2021, (23) United States**	First-line advanced NSCLC, CheckMate 227 and CheckMate 9LA	PD-L1 ≥ 1%, PD-L1 < 1%	Nivo(3 mg/kg Q2W) + Ipi (1 mg/kg Q6W) and Nivo (360 mg Q3W) + Ipi (1 mg/kg Q6W) + 2 cycles of chemo	Chemo	Lifetime Horizon	US Healthcare Perspective	Nivo + Ipi $110,333 Nivo + Ipi + Chemo: $217,820	Nivo + Ipi: 0.46, Nivo + Ipi + Chemo: 0.59	Nivo + Ipi: $239,072 per QALY gained Nivo + Ipi + Chemo $838,198 per QALY gained	Nivo + Ipi or Nivo + Ipi plus chemo was not cost-effective regardless of PD-L1 expression levels.

NSCLC, non-small cell lung cancer; PD-L1, programmed cell death ligand-1; Nivo, Nivolumab; Ipi, Ipilimumab; Chemo, Chemotherapy; QALY, Quality-adjusted life years; US, United States; QnW, every n weeks; mg/kg, milligram per kilogram; WTP, willingness-to-pay; TMB, Tumor mutational burden; ΔCosts/ΔQALY, Incremental costs per incremental quality adjusted life years.

Chemotherapy in the CheckMate 227 trial: platinum-doublet chemotherapy (every 3 weeks for up to four cycles) Chemotherapy in the CheckMate 9LA trial: carboplatin plus paclitaxel for patients with squamous histology or carboplatin plus pemetrexed or cisplatin plus pemetrexed for patients with non-squamous histology.

### 2.4. Quality assessment of the methodology

The quality assessment of the included studies was performed by using the Philips checklist (14) and the Consensus Health Economic Criteria (CHEC) checklist (15). The quality of the methodology was assessed by one reviewer and validated by a second reviewer. Any issues of discrepancy were resolved through consensus or by consultation with a third reviewer.

## 3. Results

**Figure 1:**
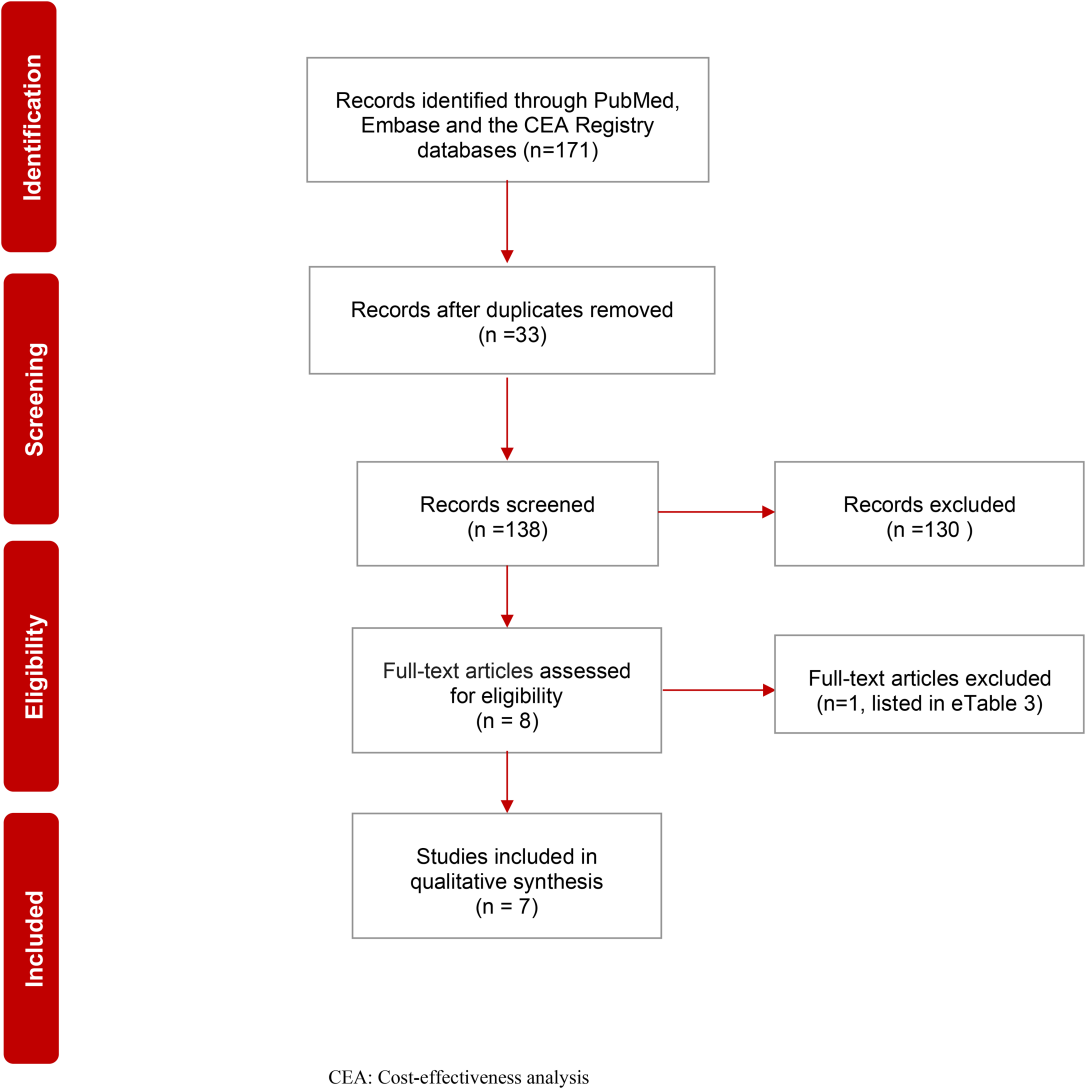
PRISMA flow chart of systematic review on the cost effectiveness of first-line nivolumab in advanced non-small cell lung cancer. CEA, Cost-effectiveness analysis.

PRISMA flow diagram (Figure 1) shows the details of all (*N* = 171) identified records. After duplicates (*N* = 33) were removed, 138 records were screened, and 130 were excluded based on title and abstract. Eight studies were then selected for full-text screening. Seven studies met the inclusion criteria and underwent data extraction. The reason for the exclusion of one study is listed in the Supplementary Appendix (Supplementary Table S2). Table 3 shows the quality assessment results based on the CHEC checklist. Table 4 shows the quality assessment results based on the Philips checklist. A schematic representation of the outcomes and differences between these checklists is presented in the Supplementary Appendix (Supplementary Figures S1, S2).

**Table 3:** Quality assessment results based on the CHEC checklist.

CHEC Checklist	Questions (1–19)																		
Study	1	2	3	4	5	6	7	8	9	10	11	12	13	14	15	16	17	18	19
**Courtney et al. (17)**	Y	Y	Y	Y	P	Y	Y	Y	Y	Y	P	Y	Y	Y	Y	Y	Y	Y	NA
**Hao et al. (18)**	Y	Y	Y	Y	P	Y	P	P	P	Y	P	P	Y	Y	Y	Y	Y	Y	NA
**Hu et al. (19)**	Y	Y	Y	Y	P	Y	Y	Y	Y	Y	Y	Y	Y	Y	Y	Y	Y	Y	NA
**Li et al. (20)**	Y	Y	Y	Y	P	Y	Y	Y	Y	Y	P	P	Y	Y	Y	Y	Y	Y	NA
**Peng et al. (21)**	Y	Y	Y	Y	Y	Y	Y	Y	Y	Y	P	P	Y	Y	Y	Y	Y	Y	NA
**Wan et al. (22)**	Y	Y	Y	Y	Y	Y	Y	Y	Y	Y	P	P	Y	Y	Y	Y	P	Y	NA
**Yang et al. (23)**	Y	Y	Y	Y	Y	Y	Y	Y	Y	Y	Y	Y	Y	Y	Y	Y	Y	Y	NA

Y, item completely fulfilled; *P*, item partially fulfilled; N, item not fulfilled; N/A, item not applicable. **Item Checklist**: 1, Study population; 2, Competing alternatives; 3, Research question; 4, Study design; 5, Time horizon; 6, Perspective; 7, Costs identified; 8, Costs measured; 9, Costs valued; 10, Outcomes identified; 11, Outcomes measured; 12, Outcomes valued; 13, Incremental analysis; 14, Costs outcomes discounted; 15, Sensitivity analysis; 16, Conclusions; 17, Generalizability of results; 18, Conflict of interest; 19, Ethical distributional issues.

**Table 4:** Quality assessment results based on the Philips checklist.

Philips Checklist: dimensions of quality																				
Study	Structure (S)									Data (D)									Consistency (C)	
	S1	S2	S3	S4	S5	S6	S7	S8	S9	D1	D2a	D2b	D2c	D3	D4a	D4b	D4c	D4d	C1	C2
**Courtney et al. (17)**	Y	Y	Y	Y	Y	Y	P	Y	P	Y	Y	Y	P	Y	Y	Y	Y	Y	Y	P
**Hao et al. (18)**	Y	Y	Y	Y	Y	Y	P	Y	P	P	P	Y	P	P	Y	Y	Y	Y	P	N
**Hu et al. (19)**	Y	Y	Y	Y	Y	Y	P	Y	Y	Y	Y	P	Y	Y	Y	Y	Y	P	N	Y
**Li et al. (20)**	Y	Y	Y	Y	Y	Y	P	Y	Y	P	P	Y	P	P	Y	Y	P	Y	N	P
**Peng et al. (21)**	Y	Y	Y	Y	Y	Y	Y	Y	P	Y	P	P	P	P	Y	Y	Y	Y	Y	N
**Wan et al. (22)**	Y	Y	Y	Y	Y	Y	Y	Y	Y	P	Y	P	P	Y	Y	Y	P	P	P	N
**Yang et al. (23)**	Y	Y	Y	Y	Y	Y	Y	Y	Y	Y	Y	Y	Y	Y	Y	Y	Y	Y	Y	N

Y, item completely fulfilled; P, item partially fulfilled; N, item not fulfilled N/A, item not applicable. **Item Checklist**: S1, Statement of decision problem/objective; S2, Justification of modelling approach; S3, Statement of scope/perspective; S4, Structural assumptions; S5, Strategies/comparators; S6, Model type; S7, Time horizon; S8, Disease states/pathways; S9, Cycle length; D1, Data Identification; D2, Pre-model data analysis; D2a, Baseline data; D2b, Treatment effects; D2c, Quality-of-life weights (utilities); D3, Data incorporation; D4, Assessment of uncertainty; D4a, methodological; D4b, structural; D4c, heterogeneity; D4d, parameter; C1, Internal consistency; C2, External consistency.

### 3.1. Included CEAs and study characteristics

In the first-line treatment of advanced NSCLC, the cost-effectiveness of nivolumab-ipilimumab and/or nivolumab-ipilimumab plus two cycles of chemotherapy was compared with standard chemotherapy. According to the PICO framework (see Table 2), in the CEAs (17–20, 22, 23) that sourced the CheckMate 227 clinical trial (*Population*), nivolumab (3 mg/kg every two weeks) in combination with ipilimumab (1 mg/kg every six weeks) (*Interventions*) was compared with platinum-doublet chemotherapy every three weeks for up to four cycles (*Comparator*) (4). In the CEAs (21, 23) that sourced the CheckMate 9LA clinical trial (*Population*), nivolumab (360 mg every three weeks) and ipilimumab (1 mg/kg every six weeks) were combined with histology-based, platinum doublet chemotherapy (every three weeks for two cycles) (*Interventions*), and were compared with chemotherapy alone (every three weeks for four cycles) (*Comparator*) (5). Study outcomes in all CEAs were expressed in incremental costs, QALYs, and ICERs (see Table 2 for details on the included study *Outcomes* and conclusions).

#### 3.1.1. Model type and health states

Markov models were developed to extrapolate study outcomes. Transition probabilities were derived from the CheckMate 227 and the CheckMate 9LA clinical trials. The methods developed by Hoyle and Henley (24) were used in studies to recreate patient data from published Kaplan-Meier Survival Curves for CEA models. In all CEAs, health states comprised stable disease (progression-free), progressed disease, and death.

#### 3.1.2. Model cycle and time horizon

Variable model cycle lengths were adapted, including intervals of one week (18), 3 weeks (21), 6 weeks (19, 20, 22, 23), and one month (17). Similarly, time horizons varied among the CEAs, including 10 years (17, 18, 20), 20 years (19), and lifetime (21–23).

*Estimation of long-term outcomes* showed variability among the included CEA studies due to: (i) variation in the extraction of data points of survival curves from the CheckMate 227 and the CheckMate 9LA trials, (ii) calibration of the probability of progressive disease to death at each model cycle (i.e., intervals of one week (18), 3 weeks (21), 6 weeks (19, 20, 22, 23), and one month (17), to fit the overall survival curve, (iii) variation in statistical techniques in fitting and extrapolating survival functions. Age-specific mortality from other causes was estimated based on the US life tables (25).

#### 3.1.3. Costs and their sources

All CEAs included the United States (US) healthcare, payer or societal perspectives, and expressed costs in US dollars (*years ranging from 2018 to 2021*). In one study (19), the authors did not specify a year for the included costs. In another study (18), the rationale for the cost year of 2018 was not included. In this study (18), the authors indicated that the vial prices of nivolumab-ipilimumab were discounted by 17%, based on a previously published study (26), and the cost of chemotherapy was $24,437 per patient regardless of histology (27). In the same study (18), the cost of maintenance chemotherapy was $5,887 for non-squamous NSCLC (27). All remaining sources for drug prices were obtained from the US Medicare and Medicaid Services (25), literature, and publicly available sources (28). Medical consumer price indices (29) was used to express costs in US dollars.

#### 3.1.4. Utility values and their sources

Health state utility estimates were based on the literature (30–33), for six CEAs (17, 18, 20–23). In one study (19), treatment-specific utilities (0.784 combination therapy and 0.693 chemotherapy) were collected in the CheckMate 227 trial (34).

#### 3.1.5. Cost-effectiveness thresholds

For the US setting, two studies used a willingness-to-pay threshold (WTP) of $100,000 per QALY (17, 18), four studies used a WTP of $150,000 per QALY (19–21, 23), and one study included both thresholds (22). In addition, one study included the perspective of the Chinese healthcare system and used a WTP of $27,351 per QALY (18).

#### 3.1.6. Cost-effectiveness results

The ICERs (cost/QALY gained) reported in the included studies which are *not deemed cost-effective* were as follows: $401,700 (healthcare perspective) (17), $434,400 (societal perspective) (21), $551,900 (received treatment up to 24 months) (17). In patients with PD-L1 < 1%, the ICER was $172,589 per QALY gained (19). In China, the ICER of nivolumab-ipilimumab was $59,773 per QALY gained, at a WTP threshold of $27,351 per QALY (18). One study (20) showed that in patients with PD-L1 ≥ 1 and PD-L1 < 1, the ICERs were $180,307 and $143,434 per QALY gained, respectively (20). One study (21) reported that the ICER was $202,275 per QALY gained (21). Another study (23), showed that the ICER of nivolumab-ipilimumab combination therapy was $239,072 per QALY compared to chemotherapy, while the ICER of nivolumab-ipilimumab plus chemotherapy compared to nivolumab-ipilimumab was $838,198 per QALY gained (23).

The ICERs reported in the included studies which are *deemed cost-effective* were as follows: In one study (19), for patients with PD-L1 expression levels ≥50% and ≥1% or a high Tumor Mutational Burden (TMB), the ICERs were $107,404 and $133,732 per QALY gained, respectively (19). In another study (18), the ICER was $75,871 per QALY gained (for the US setting). However the credibility of the data sources in this study (18) is questionable and poses a challenge to accurately compare study outcomes. For the US setting, the outcomes of the above mentioned study (18) should be interpreted with caution. Another study (22) reported that the ICER was $104,238 per QALY gained (regardless of the PD-L1 expression level) (22).

#### 3.1.7. Sensitivity and/or subgroup analyses

For patients with PD-L1 levels <1%, ≥1% and ≥50%; the ICERs were $332,100, $440,100 and $375,700 per QALY gained, respectively (17). The most influential model inputs were drug acquisition costs, duration of combination immunotherapy, patients' body weight and survival hazard ratio. In one study (19), the analysis on patients with a high TMB, resulted in an ICER of $69,182 per QALY gained compared with chemotherapy. In this study (19), patients with PD-L1 < 1%, nivolumab-ipilimumab combination therapy could be deemed cost-effective, if the cost of nivolumab were to be discounted by 21% or the cost of ipilimumab were to be discounted by 24% (19). Another study (20) reported from the US perspective that the ICERs were $143,434, $196,507 and $212,111 per QALY gained, in patient with PD-L1 < 1, ≥1, and ≥50%, respectively (20). The authors in this study calculated that the cost of nivolumab should be discounted by 20% in order to have an ICER below the WTP threshold (20). In one study (22), the authors showed when patients' weight increased to 140 kg or the overall survival hazard ratio increased to 0.84, the ICER was above the WTP threshold of $150,000 per QALY (22). Finally, one study (21) showed that patients with Eastern Cooperative Oncology Group score of 0 and central nervous system metastases favored nivolumab-ipilimumab plus chemotherapy, with more than a 50% probability of being cost-effective compared with chemotherapy (21). However, the cost-effectiveness probability was extremely low for subgroups of patients with unfavorable HR of overall survival, such as those older than 75 years, with squamous NSCLC, and liver metastases (21). In this study, when the cost of nivolumab was reduced by at least 28%, nivolumab-ipilimumab plus chemotherapy was cost-effective compared with chemotherapy alone at a threshold of $150,000 per QALY (21).

### 3.2. Methodological quality assessment of the included cost-effectiveness studies

Table 3 shows methodological quality assessment results based on the CHEC checklist. The CHEC checklist consists of 19 questions (15). The quality outcomes of each study were based on whether insufficient or missing information was identified in the article, or in other published materials. If the study authors paid sufficient attention to the listed checklist items then the assessment criteria were fulfilled. Table 4 shows the quality assessment results based on the Philips checklist. This checklist consists of 20 quality dimensions, according to model structure, data, and consistency (14). Similar assessment criteria were employed, and the quality outcomes of each study based on the Philips checklist are presented in Table 4. For a visual representation of the quality assessment study findings and differences among these checklists, see the Supplementary Appendix (Supplementary Figures S1, S2).

Based on the CHEC checklist, time horizon and health outcome measurement (Table 3) were items that were “partially fulfilled” by Courtney et al. (17). In studies reported by Hu et al. (19), Hao et al. (18), Li et al. (20), Wan et al. (22), and Peng et al. (21), a combination of “partially fulfilled” and “not reported” checklist items affected the quality of each study. Using this checklist, the CEA that scored the highest methodological quality was published by Yang et al. (23).

Based on the Philips checklist, time horizon, cycle length, health utilities, and external consistency (Table 4) were items that were “partially fulfilled” by Courtney et al. (17). In studies reported by Hu et al. (19), Hao et al. (18), Li et al. (20), Wan et al. (22), and Peng et al. (21), a combination of “partially fulfilled” and “not reported” checklist items affected the quality of each study. According to the Philips checklist, the CEA that scored the highest methodological quality was published by Yang et al. (23).

Overall, our assessment highlighted shortcomings in data identification and methods of transparency. Quantification of health state utility values, estimation of drug costs, the accuracy of data sources, and their credibility have important quality implications on the cost-effectiveness outcomes. None of the included studies fulfilled all of the criteria reported in the Philips and the CHEC checklists. Although the conclusions of the four CEAs indicated that nivolumab-ipilimumab combination therapy had favorable cost-effectiveness (i.e., 4 out of 7 studies), the quality assessment of these studies revealed that there were a number of uncertainties and limitations pertaining to each study. From a clinical perspective, Ipilimumab has no approved single-agent (monotherapy) activity in the treatment of NSCLC, and its mechanism of action (i.e., synergy or additivity), when combined with nivolumab, is not fully understood in this setting (35). To compound the economic consequences presented in these limited number of CEAs, ipilimumab's drug action as a combination therapy poses significant uncertainty and requires further clinical investigation (35). We encourage further research to address the economic consequences of these combination agents in future CEAs and clinical uncertainties of ipilimumab for NSCLC in future trials.

## 4. Discussion

Nivolumab-ipilimumab combination therapy has a high price tag, and the potential to be used for a range of indications, also in combination with other agents. Our systematic review showed that the methods of estimation of long-term outcomes, quantification of the health state utility, estimation of drug costs, the accuracy of data sources, and their credibility have important implications on the ICERs. None of the included studies fulfilled all of the requirements presented in the Philips checklist, and the CHEC checklist. Quality assessment of the included studies highlighted shortcomings in data identification, uncertainty assessment, and methods transparency domains.

The estimation of long-term immunotherapy outcomes has important implications. Given that the CEA model inputs were sourced from the clinical trials, the durability of response, and potential long-term survival after immunotherapy are crucial factors for these economic analyses. Currently, the minimum effective dose of immunotherapy remains unknown, as does the optimal duration of treatment. A better understanding of optimal drug dosage and treatment duration may influence the overall costs of immunotherapy. To theoretically address the long-term estimation of outcomes, CEAs are encouraged to vary nivolumab-ipilimumab dosing and treatment duration in their sensitivity analyses.

Assessing the cost-effectiveness of immunotherapy drugs depends not only on the relative efficacy of treatments observed in the clinical trials, but also on the model structure, and assumptions. Good practice recommendations were developed specifically for fitting curves to observe progression-free and overall survival (36, 37). Although stochastic uncertainty (i.e., model parameters, and assumptions) is usually assessed in CEA models, structural uncertainty (i.e., alternative modeling approaches) is not often considered. It is common practice to acknowledge potential limitations in model structure, however, identified studies in our review lack clarity about methods to characterize the uncertainty surrounding alternative structural assumptions and their contribution to decision uncertainty. Given that alternative modeling techniques (i.e., cure models, spline-based models) may complement standard methods, future CEAs may incorporate structural uncertainty by considering alternative modeling approaches concurrently.

Although patient-reported outcomes (PROs) were collected in the CheckMate 227 and the CheckMate 9LA trials, six CEA models were developed based on health utility estimates that were sourced from previously published studies (30–33). Similarly, utility decrements of AEs were sourced from the publicly available literature. Cancers with a high TMB, such as NSCLC, are associated with higher immune-related AEs (irAEs) during immunotherapy treatment, suggesting that these cancers are associated with a higher risk of irAEs than cancers with a low TMB. Although irAEs are rare, the cost of treatment in such cases is rather high. Therefore, the benefits of nivolumab-ipilimumab combination therapy could be over- or underestimated in the included models. The inclusion of irAEs in future economic models of NSCLC is encouraged.

TMB is an emerging biomarker for immunotherapy in lung cancer (38–42). The results of the CheckMate 568 showed the TMB of more than 10 mutations per mega base could be used as an effective cutoff value for selecting responders (43). Similarly, the analysis of Hellmann et al. showed that the first line treatment with nivolumab-ipilimumab provided clinical benefits for patients with NSCLC with a high TMB (≥10 mutations per mega base), regardless of their tumor PD-L1 expression levels (44). Although nivolumab-ipilimumab provided the greatest absolute survival for patients with a high TMB in the CheckMate 227 trial, the clinical benefits were similar to those of chemotherapy in patients regardless of their TMB. Therefore, it is necessary to understand the implications of TMB as a biomarker and then re-analyze clinical and cost-effectiveness findings accordingly.

This study is the first systematic review that focused on the methodological quality of CEAs conducted specifically for the front-line nivolumab-ipilimumab combination. Previously published systematic reviews of CEAs focusing on immunotherapy in advanced NSCLC (45–47), did not assess the quality of the study methodology based on either the Philips checklist or the CHEC checklist. One study (45) used the Consolidated Health Economic Evaluation Reporting Standards checklist (48). However, this checklist is not designed for the quality assessment of CEA study methodology.

All in all, efficient allocation of existing resources is essential for health systems to meet the evolving needs of populations and sustainability efforts. From our analysis, the quality assessment of the included CEAs highlighted shortcomings in various domains of the included checklists. To improve methodological study quality, we encourage authors of future CEAs to consider the inclusion of either the CHEC or the Philips checklist in their studies and to follow its guidance to report their analyses. The application of high-quality knowledge that stems from scientific evidence and economic modeling can aid in achieving sustainable health systems worldwide. Improving the methodological quality of the future CEAs would be a significant step in the right direction toward this achievement.

## Data Availability

The original contributions presented in the study are included in the article/**Supplementary Material**, further inquiries can be directed to the corresponding author/s.
