# Supplementary material for: Cost-effectiveness of first line nivolumab-ipilimumab combination therapy for advanced non-small cell lung cancer: A systematic review and methodological quality assessment
Source: Front Health Serv. 2023 Mar 13;3:1034256. doi: 10.3389/frhs.2023.1034256 (PMC10012633; doi:10.3389/frhs.2023.1034256)
Supplement: Supplementary file 1 [file Datasheet1.docx]

**Supplementary Appendix**

**Table 1:** Search strategy

*a. Search strategy:* PubMed (MEDLINE)

| Population | ("non-small-cell lung cancer"[Mesh] OR NSCLC*[tiab]) |
| --- | --- |
| Intervention | ("Nivolumab"[Mesh] OR PD1*[tiab] OR checkpoint*[tiab]) AND ("Ipilimumab"[Mesh] OR CTLA-4*[tiab]) AND (first line*[tiab] OR front line*[tiab] OR treatment naïve*[tiab]) |
| Comparator | No search string |
| Outcomes | No search string |
| Limits | *Study design:*  (“cost-effectiveness analysis”[Mesh] OR “economic”[Mesh] OR “economic evaluation”[tiab] OR economic value*[tiab]) |
| Limits | *Publication period:* No restrictions |
|  | *Language:* No restrictions |

*b. Search strategy:* EMBASE

| Population | ('non-small cell lung cancer'/exp OR NSCLC*:ti,ab) |
| --- | --- |
| Intervention | ('Nivolumab'/exp OR checkpoint*:ti,ab OR PD1*:ti,ab) AND ('Ipilimumab'/exp OR CTLA-4*:ti,ab) AND (first line:ti,ab OR front line:ti,ab OR treatment naive:ti,ab) |
| Comparator | No search string |
| Outcomes | No search string |
| Limits | *Study design:* ('cost effectiveness /exp OR 'cost utility'/exp OR 'economic'/exp OR 'economic evaluation'/de OR (economic NEAR/3 value*):ti,ab) |
| Limits | *Publication period:* No restrictions |
|  | *Language:* No restrictions |

*c. Search strategy:* The Cost-Effectiveness Analysis Registry

| Database | The CEA registry  [http://healtheconomics.tuftsmedicalcenter.org/cear2n/search/search.aspx] |
| --- | --- |
| Population | (non-small cell lung cancer) OR (NSCLC) |
| Intervention | (Nivolumab) OR (Ipilimumab) AND (first line) |
| Comparator | No search string |
| Outcomes | No search string |
| Limits | No limits |

**Abbreviations** CEA: Cost effectiveness analysis, NSCLC: non-small cell lung cancer

**Table 2: Excluded full-text studies**

| Reference | Reason for exclusion |
| --- | --- |
| Teng MM, Chen SY, Yang B, et al. Determining the optimal PD-1/PD-L1 inhibitors for the first-line treatment of non-small-cell lung cancer with high-level PD-L1 expression in China. Cancer Med. 2021;10(18):6344-6353. doi:10.1002/cam4.4191. | Clinical data for nivolumab were sourced from the CheckMate 026 clinical trial, instead of the CheckMate 227 and/or CheckMate 9LA trials. The US FDA did not approved first-line nivolumab based on the CheckMate 026 trial. |

**Figure 1.** Quality assessment of the study methodology by the CHEC checklist

**
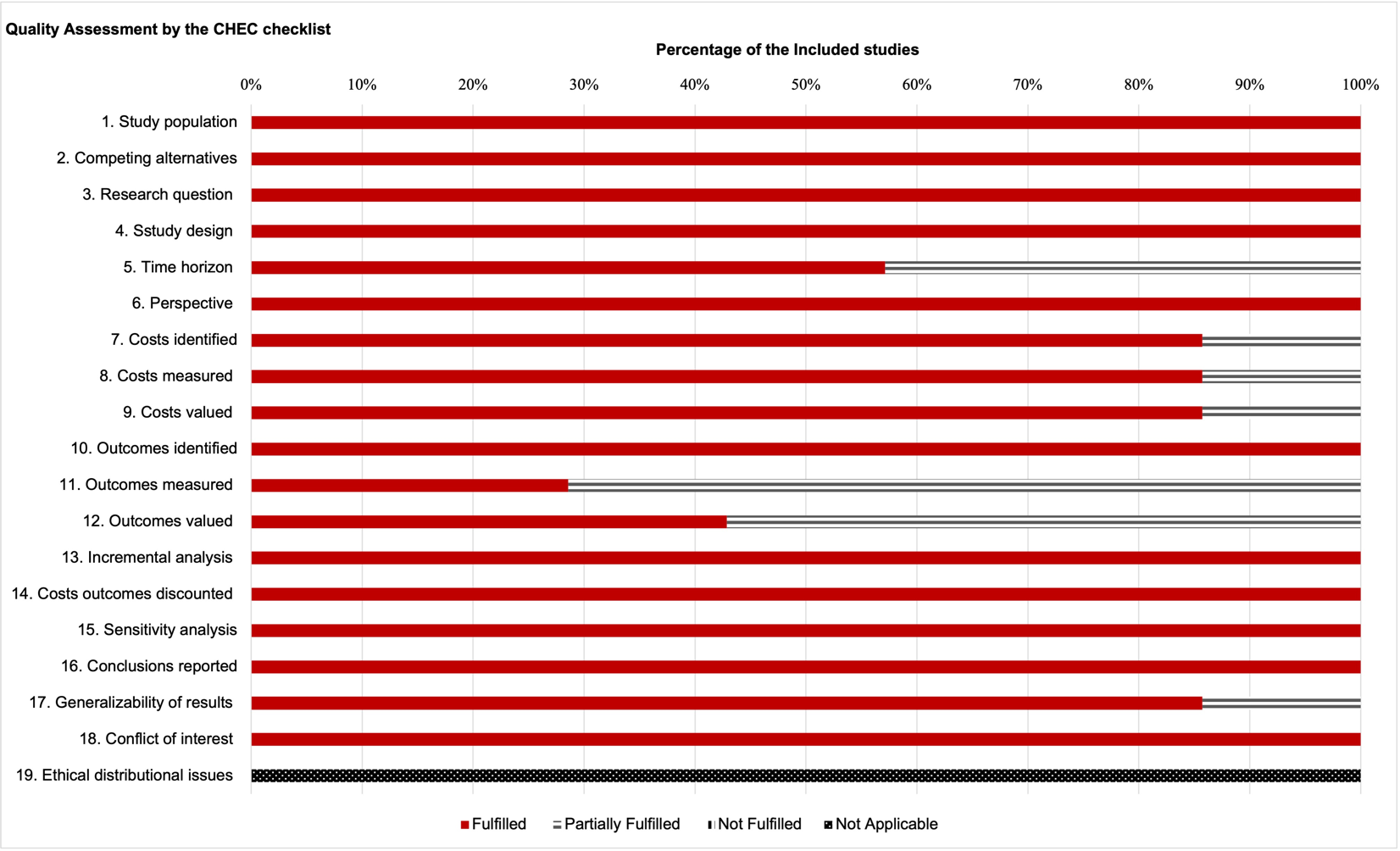
**

F; item completely fulfilled, P; item partially fulfilled, N; item not fulfilled, N/A; item not applicable

**Item Checklist:** 1; Study population, 2; Competing alternatives, 3; Research question, 4; Study design, 5; Time horizon, 6; Perspective, 7; Costs identified, 8; Costs measured, 9; Costs valued, 10; Outcomes identified, 11; Outcomes measured, 12; Outcomes valued, 13; Incremental analysis, 14; Costs outcomes discounted, 15; Sensitivity analysis, 16; Conclusions, 17; Generalizability of results, 18; Conflict of interest, 19; Ethical distributional issues

**Figure 2.** Quality assessment of the study methodology by the Philips checklist

**
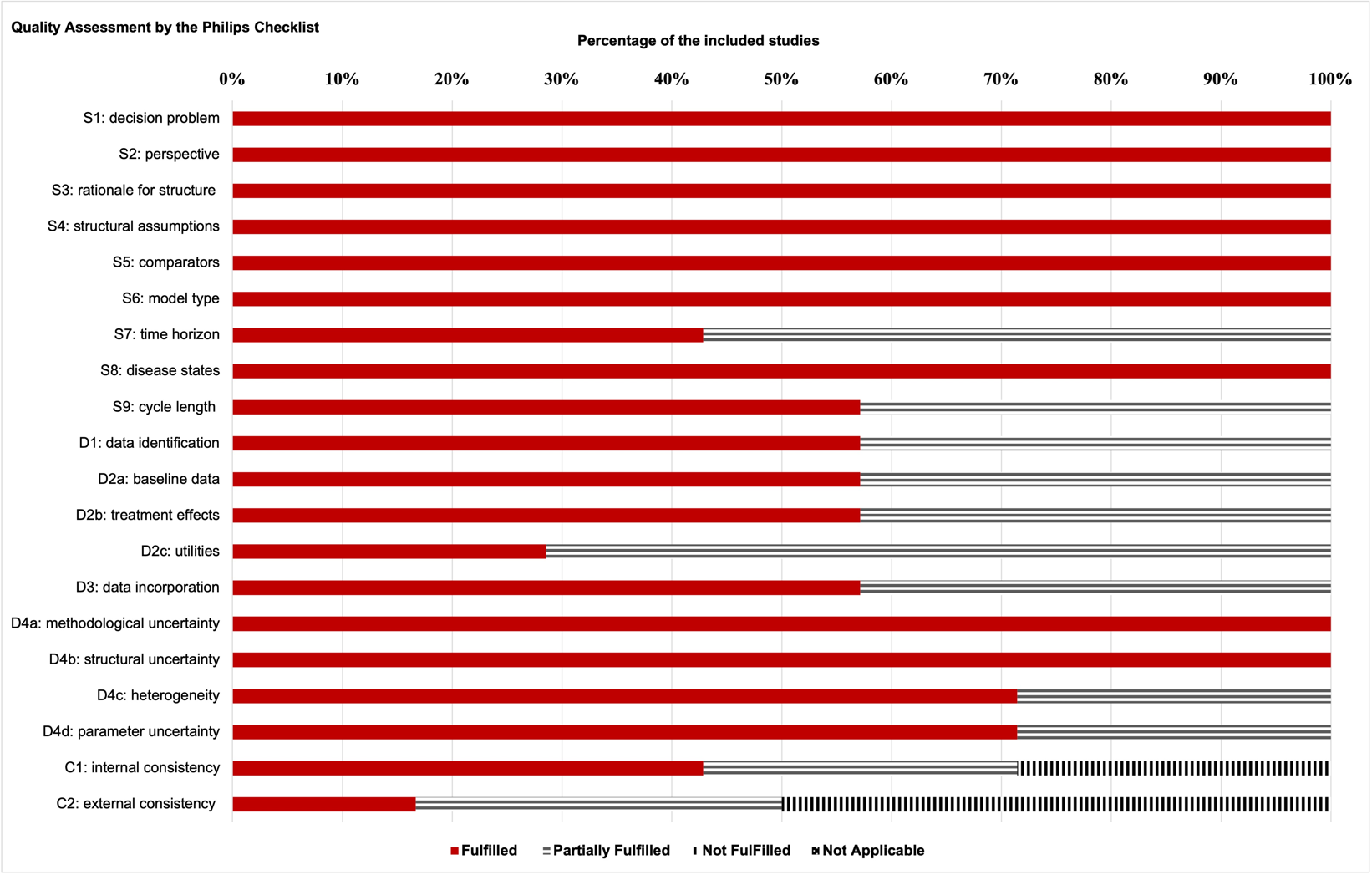
**

F; item completely fulfilled, P; item partially fulfilled, N; item not fulfilled, N/A; item not applicable

**Item Checklist:** S1; Statement of decision problem/objective, S2; Justification of modelling approach, S3; Statement of scope/perspective, S4; Structural assumptions, S5; Strategies/comparators, S6; Model type, S7; Time horizon, S8; Disease states/pathways, S9; Cycle length, D1; Data Identification, D2; Pre-model data analysis, D2a; Baseline data, D2b; Treatment effects, D2c; Quality-of-life weights (utilities), D3; Data incorporation, D4; Assessment of uncertainty, D4a; methodological, D4b; structural, D4c; heterogeneity, D4d; parameter, C1; Internal consistency, C2; External consistency
